# Light-Assisted Advanced Oxidation Processes for the Elimination of Chemical and Microbiological Pollution of Wastewaters in Developed and Developing Countries

**DOI:** 10.3390/molecules22071070

**Published:** 2017-06-26

**Authors:** Stefanos Giannakis, Sami Rtimi, Cesar Pulgarin

**Affiliations:** SB, ISIC, Group of Advanced Oxidation Processes (GPAO), École Polytechnique Fédérale de Lausanne (EPFL), Station 6, CH-1015 Lausanne, Switzerland; sami.rtimi@epfl.ch

**Keywords:** urban and hospital wastewater, urine treatment, pathogen microorganisms, emerging contaminants, UV/H_2_O_2_, photo-Fenton, *E. coli*, MS2 coliphage, saccharomyces cerevisiae, micropollutants

## Abstract

In this work, the issue of hospital and urban wastewater treatment is studied in two different contexts, in Switzerland and in developing countries (Ivory Coast and Colombia). For this purpose, the treatment of municipal wastewater effluents is studied, simulating the developed countries’ context, while cheap and sustainable solutions are proposed for the developing countries, to form a barrier between effluents and receiving water bodies. In order to propose proper methods for each case, the characteristics of the matrices and the targets are described here in detail. In both contexts, the use of Advanced Oxidation Processes (AOPs) is implemented, focusing on UV-based and solar-supported ones, in the respective target areas. A list of emerging contaminants and bacteria are firstly studied to provide operational and engineering details on their removal by AOPs. Fundamental mechanistic insights are also provided on the degradation of the effluent wastewater organic matter. The use of viruses and yeasts as potential model pathogens is also accounted for, treated by the photo-Fenton process. In addition, two pharmaceutically active compound (PhAC) models of hospital and/or industrial origin are studied in wastewater and urine, treated by all accounted AOPs, as a proposed method to effectively control concentrated point-source pollution from hospital wastewaters. Their elimination was modeled and the degradation pathway was elucidated by the use of state-of-the-art analytical techniques. In conclusion, the use of light-supported AOPs was proven to be effective in degrading the respective target and further insights were provided by each application, which could facilitate their divulgation and potential application in the field.

## 1. Problems Related with Presence of Microorganisms and Micropollutants 

Wastewater treatment plants (WWTPs) have been built, transformed and updated through the years to effectively prevent solids, organic and inorganic compounds (carbon, nitrogen, phosphorus, etc.) and more, which enter the environment. The challenge posed by the pollutants is a matter that the majority of the WWTPs are not equipped to handle. The micropollutants are (in a high percentage) invulnerable to biological treatment; the transfer from source to the environment is therefore facilitated, leading to further accumulation in the environment; their presence has been associated with minor and major health risks, toxicity and more [1,2]. 

The risk of microorganisms’ presence in natural water bodies is more explored compared to micropollutants [3,4]. Water scarcity has led to reuse concepts and many countries worldwide have included legislation concerning the removal thresholds according to the subsequent water reuse, e.g., in Italy, [5]. However, chlorination is still the most widespread technique, with its inherent problems, such as disinfection by-products (e.g., trihalomethanes) formation, and where funds are available UV has been applied. 

The problem of microorganisms’ occurrence in water sources has been identified for many decades. Water related diseases plague the developing countries using compromised drinking water sources [6], or reusing wastewater for food production [7], but also many outbreaks have occurred by cross contamination of public access waters in the developed states [8]. Apart from the notorious diseases such as cholera, gastro-enteritis or dengue fever, the contamination of water sources poses risks to populations which have incorporated their use in their economical-related activities, such as fishing or other economic activities. 

The main problem associated with the occurrence of micropollutants (MPs) in the environment is the lack of information, concerning the side-effects of their presence in the receiving matrix [9]. For instance, little is known on the long term effects of pollutants, classified as “potentially not harmful”; there are no studies on the accumulation or the chronic toxicity of such substances in plants, animals, or humans. There is a relatively long list of MPs actually found in the environment, which derived from the various anthropogenic activities [10,11]. To date, not all substances have been assessed on their potential actions against the environment.

Furthermore, toxicologically speaking, most of the substances that have been assessed for their risks, have been directed through single-compound investigations, when the reality differs significantly [12]. The environment contains a mixture of a vast number of compounds, which could even react with each other, affecting their mode of action [13]. The result has been demonstrated to be severe, with cases reporting compounds that had no prior effect on species, to demonstrate harmful properties when found in mixtures [14,15,16]. Apart from the toxicity problems, other critical drug-related issues that have emerged over the years are: (i) the enhancement of antibiotic resistance, by the presence of antibiotics and their metabolites in the environment [17]; (ii) the problematic identification of the transformed metabolites of drugs [18]; and (iii) the bioaccumulation of pollutants in living organisms in the environment.

To better suggest treatment goals and methods, this work will address the use of Advanced Oxidation Processes (AOPs) as a greener and sustainable disinfection and decontamination method of hospital and urban wastewater in both developed and developing countries. Each context will be treated with regard to its specific issues, dealing with the economic feasibility and technical applicability of the various light-assisted AOPs. The UV-based ones will form the basis of treatment for developed countries whereas the photo-Fenton process will be presents as a viable solution for the sunny developing countries around the globe. After contextualization of the problems micropollutants (MPs) and microorganisms (MOs) cause to the environment and to humans, details will be provided on their presence, the contribution of hospitals in aggravating the actual situation and an overview of the results obtained in the different environments by the various AOPs.

## 2. Hospital Wastewater Effluents and Types and Pollutants

### 2.1. Hospital Wastewater and Micropollutants

Hospital wastewater (HWW) is the result of the residue collection from the various water-consuming activities taking place within its premises. These services include [19]:

(i) Human sewage
(ii) Kitchen and laundry
(iii) Heating and cooling processes
(iv) Laboratorial discharge (clinics and research centers)
(v) Wards and outpatients contribution

The first categories are common also in municipal wastewater (MWW), which is the reason that led practitioners to suggest co-treatment in MWWTPs, sometimes with only a pre-treatment (e.g., chlorination) [20], only to limit the microbiological-related risk. The reality suggests that within HWW there are many substances, such as disinfectants, organic compounds, therapeutic metals, rare microbial agents or antibiotic-resistant ones [21,22,23], often in high concentrations that modify significantly the composition of HWW compared to MWW. For this reason, many authors have openly objected to the co-treatment practice so far [24,25,26]. 

The presence of Pharmaceutically Active Compounds (PhACs) in hospital wastewater is a hot topic, with a variety of works dedicated to the characterization of their nature [19,27,28,29,30] and their importance in the overall load [18]. The characteristics of HWW are influenced by a variety of factors, such as the size of the hospital, the range of services and activities, the season of the year, the time of the day [19,31], and more. Table 1 presents an overview comparing HWW with MWW, in terms of a single unit (patient/inhabitant). 

It is obvious that the composition of either micro- or macropollutants in each case is significantly different (see Table 1), indicating one of the reasons for failure of treatment by conventional WWTPs. The micropollutants arriving in WWTPs are of a range of μg or ng, and also are reported to affect the nature of the WW in the treatment plant (solubility, volatility, adsorbability, absorbability, biodegradability, polarity, and stability) [31]. 

For the pre-mentioned reasons, HWW should be treated as a separate entity [31]. The economic and overall risks should be assessed [25], on-site treatment should be implemented as close as possible to the source [32,33], the consideration of no-mix toilets for urine separation must be taken into account [34] and reducing the quantities can considerably mitigate the effluent amounts if direct discharge in surface water is expected.

### 2.2. Hospital Wastewaters and Microorganisms

Similar to MPs, HWW are carriers of higher microbiological agent loads, compared to their urban counterparts. In principle, the MWW are subjected to higher dilution and the intrinsic properties of HWW imply the presence of higher and possibly more infectious agents. In a recent review [35] the differences between MWW and HWW effluents in various countries have been presented, with compelling variations in the distribution of microorganisms’ species and quantities. This microorganism load is usually led to co-treatment with MWW in MWWTPs and their removal efficiency is a function of the existing treatment. Among others mentioned before, some authors [36] stressed the importance of treating HWW separately, on-site, to effectively reduce micro- and macropollutants, but also stressing the need for microorganisms’ elimination. Problematic treatment or inexistent treatment can lead to the problems mentioned before (antibiotic resistance, bioaccumulation, etc.) as well as directly jeopardize the drinking water sources in developing countries [37]. 

## 3. Light-Assisted AOPs and Their Action in Chemical and Microbiological Pollutants’ Degradation

Due to the recalcitrant nature of many existing MPs, the existing biological and physicochemical treatment methods have been proven unable to efficiently degrade them in WWTPs [2]. Subsequently, their elimination relies on further, “quaternary” treatment, such as advanced oxidation treatment. These processes have successfully mineralized or converted the persistent MPs to less harmful forms [33]. AOPs can be used as pre-treatment or post-biological treatment processes. Depending on the target, they can achieve conversion of recalcitrant pollutants to biodegradable ones, or act as a polishing step. In the respective cases, the residence time in biological treatment is reduced or the residual pollutant content can be eliminated [38,39,40,41].

Ozonation and AOPs are redox technologies with main characteristic the non-selectivity on the target and share the production of the highly oxidative hydroxyl radical (HO^●^) [2]. After fluorine, the HO^●^ is the second most powerful oxidant (3.03 eV, compared to 2.80), with reaction rates ranging from 10^−6^ to 10^−9^ M^−1^ s^−1^ [42]. 

The AOPs typically involve chemical agents (metals, ozone or hydrogen peroxide) and an assistive energy source, such as UV or visible light, current, ultrasound or γ-irradiation [43]. Some examples of AOPs are:

- **Ozone-based**: O_3_/H_2_O_2_, O_3_/UV, O_3_/UV/H_2_O_2_
- **UV-based**: UV, UV/H_2_O_2_
- **Fenton**-**related**: (Fe/H_2_O_2_), including photo-Fenton, electro-Fenton, etc.
- **Heterogeneous photocatalysis**, such as (TiO_2_/hv)
- **γ-radiolysis**
- **Ultrasound-based**: sonolysis, ultrasound-supported Fenton, etc.

Although the hydroxyl radical is the main oxidizing agent in these processes, their application often induces the production and participation of other reactive oxygen species (ROS), such as superoxide radical anions, hydroperoxyl radicals, singlet and triplet oxygen, etc. [33,43]. Another main advantage of the AOPs application is the characteristic versatility with which the method can be achieved. For instance, photolysis acts directly or indirectly, by absorption of energy and excitation or photosensitizing agents, typically dissolved organic matter (DOM) [42]. 

### 3.1. UV-Based Processes (UV, UV/H_2_O_2_)

UV treatment consists on the direct photo-transformation of organic compounds. In UV direct photolysis, the micropollutant must absorb the incident radiation and undergo degradation starting from its excited state. This treatment has been the most known and widely used irradiation method in initiating oxidative degradation processes. Some organic pollutants effectively absorb UV-C light directly, and absorption of this high-energy can cause destruction of the chemical bonds and subsequent breakdown of the contaminant.

The main factors which will affect the degradation of MPs in the UVC light assisted UV/H_2_O_2_ processes are the UV absorption and its quantum yield [44]. The molar absorption coefficient, i.e., UV absorption is an indication of the strength with which a molecule absorbs UV, and consequently, cause its degradation [42,45,46]. In principle, reaction kinetics of the organic substrate with the oxidant are described by second order law, as follows Equation (1):

(1) $$r\left(-M\right)=\frac{d{\left[M\right]}_{t}}{dt}=\;k_{oxidant,M}\left[M\right]\left[oxidant\right],$$

where r(-M) represents the rate of degradation of the MP. At the same time, direct photolysis contributes in the dual manner mentioned before, provided that other WW constituents and physicochemical characteristics are present, such as pH conditions [33]. Other important factors include the concentration of the target compound, the pH of the matrix, the amount of H_2_O_2_, the presence/absence of scavenging compounds (e.g., bicarbonates) and the reaction time. 

UV/H_2_O_2_ treatment is considered an advanced oxidation process because it involves the generation of hydroxyl radicals (HO•) produced by homolytic cleavage of hydrogen peroxide. Photolysis of H_2_O_2_ yields two HO• radicals per photon absorbed. The hydroxyl radicals are strong oxidants (E° = 2.8 V) with fast reactivity due to their non-selectiveness. The efficiency of the process will depend strongly on the HO• production velocity. The most basic initiation, propagation and termination reactions with main components in water are as follows (Equations (2)–(12)) [47]:

(2) $$H_{2}O_{2}\overset{hv}{\to }\;2\;HO^{•}$$

(3) $$H_{2}O_{2}+\;HO^{•}\to HO_{2}^{•}\;+\;H_{2}O$$

(4) $$H_{2}O_{2}+O_{2}^{•-}\to \;HO^{•}+\;O_{2}+OH^{-}$$

(5) $$HO^{•}+O_{2}^{•-}\to O_{2}+OH^{-}$$

(6) $$HO^{•}+targets\to Products$$

(7) $$HO^{•}+DOC/NOM\to Products$$

(8) $$HO^{•}+CO_{3}^{-}\to CO_{3}^{•-}+OH^{-}$$

(9) $$HO^{•}+HCO_{3}^{-}\to CO_{3}^{•-}+H_{2}O2$$

(10) $$2\;HO^{•}\to \;H_{2}O_{2}$$

(11) $$2\;HO_{2}^{•}\to H_{2}O_{2}+\;O_{2}$$

(12) $$HO^{•}+\;HO_{2}^{•}\to O_{2}+H_{2}O$$

The molar absorption coefficient of H_2_O_2_ is only 18.7 M^−1^·cm^−1^ at 254 nm [48]. Hence, the efficiency of UV/H_2_O_2_ process decreases drastically with the presence of strong photon absorbers or when the UV absorbance of the target pollutant is high.

### 3.2. Fenton-Related Reactions (Fenton, Photo-Fenton, Solar Light)

The Fenton process is an attractive oxidative system for water and wastewater treatment, due to iron abundance in nature and low inherent toxicity, as well as the fact that hydrogen peroxide is easy to handle and environmentally safe, decomposing spontaneously to H_2_O and O_2_.

It has been demonstrated that Fenton’s reagent is able to destroy toxic compounds in wastewater [49]. Production of HO^•^ by the Fenton reagent takes place by addition of H_2_O_2_ to Fe^2+^ salts trough the following reactions [50,51]. “R” is used to describe the reacting organic compound (Equations (13)–(34)) [52]:

(13) $$Fe^{3+}+H_{2}O↔Fe{\left(OH\right)}^{2+}+H^{+}$$

(14) $$Fe^{3+}+2H_{2}O↔Fe{\left(OH\right)}_{2}^{+}+2H^{+}$$

(15) $$2Fe^{3+}+2H_{2}O↔Fe_{2}{\left(OH\right)}_{2}^{4+}+2H^{+}$$

(16) $$Fe^{3+}+\;H_{2}O_{2}↔\;Fe^{3+}{\left(HO_{2}\right)}^{2+}+H^{+}$$

(17) $$Fe{\left(OH\right)}^{2+}+H_{2}O_{2}↔Fe^{3+}\left(OH\right){\left(HO_{2}\right)}^{+}+H^{+}$$

(18) $$Fe^{3+}{\left(HO_{2}\right)}^{2+}\to Fe^{2+}+HO_{2}^{•}$$

(19) $$Fe^{3+}\left(OH\right){\left(HO_{2}\right)}^{+}\to Fe^{2+}+HO_{2}^{•}+OH^{-}$$

(20) $$Fe^{2+}+H_{2}O_{2}\to Fe^{3+}+\;HO^{•}+OH^{-}$$

(21) $$Fe^{2+}+\;HO^{•}\to Fe^{3+}+OH^{-}$$

(22) $$HO^{•}+H_{2}O_{2}\to HO_{2}^{•}+H_{2}O$$

(23) $$Fe^{2+}+HO_{2}^{•}\to Fe^{3+}{\left(HO_{2}\right)}^{2+}$$

(24) $$Fe^{2+}+O_{2}^{•-}+H^{+}\to Fe^{3+}{\left(HO_{2}\right)}^{2+}$$

(25) $$Fe^{3+}+HO_{2}^{•}\to Fe^{2+}+O_{2}+H^{+}$$

(26) $$Fe^{3+}+O_{2}^{•-}\to Fe^{2+}+O_{2}$$

(27) $$HO_{2}^{•}\to O_{2}^{•-}+H^{+}$$

(28) $$O_{2}^{•-}+H^{+}\to HO_{2}^{•}$$

(29) $$HO_{2}^{•}+HO_{2}^{•}\to H_{2}O_{2}+O_{2}$$

(30) $$HO_{2}^{•}+O_{2}^{•-}+H_{2}O\to H_{2}O_{2}+O_{2}+OH^{-}$$

(31) $$HO^{•}+HO_{2}^{•}\to H_{2}O+O_{2}$$

(32) $$HO^{•}+O_{2}^{•-}\to O_{2}+OH^{-}$$

(33) $$HO^{•}+HO^{•}\to H_{2}O_{2}$$

(34) $$HO^{•}\;+\;RH\;\to \;H_{2}O\;+\;R^{•}$$

However, exposure to light enhances the Fenton reaction by the photo-regeneration of Fe (II), when reducing Fe (III) or via a ligand to metal charge transfer (LMCT). Hence, there is a double production of hydroxyl radicals (L is an organic ligand) [53] (Equations (35) and (36)):

(35) $${\left[Fe^{3+}-L\right]}^{3+}+2H_{2}O\;\overset{hv\;\left(LMCT\right)}{\to }\;{\left[Fe{\left(H_{2}O\right)}_{2}\right]}^{2+}+L^{•+}$$

(36) $${\left[Fe{\left(H_{2}O\right)}_{5}\left(OH\right)\right]}^{3+}\overset{hv}{\to }Fe^{2+}+\;H^{+}+HO^{•}$$

Thus, photo-Fenton is a process that is able to use solar radiation as input taking advantage not only of the UV portion contained in solar radiation but also because of the ability of some compounds such as Fe^3+^-hydroxy and Fe^3+^-acid to absorb energy in the visible spectra. 

The use of solar light as source of radiation for activating the hydroxyl radicals is not a new concept: several researches have proven the efficiency of solar light as an activating agent for the Fenton reaction. In this process, the Fe^2+^ is continuously recycled, reducing the amount of iron salts required (and their further disposal) for the Fenton’s reaction. This feature makes the photo-Fenton process more applicable and attractive for application in sunny regions around the globe, as reviewed extensively in [54,55]. 

## 4. Problem Identification and Contextualization: Micropollutants and Microorganisms in Developed and Developing Countries

Since micropollutants have been identified in many cases as high risk compounds, many works have been initiated to identify their presence in the environment [2,56]. Moving in backward steps, the presence in environmental water matrices is a result of a variety of pathways. One of the main sources, which will be further analyzed later on, are the MWWTPs, due to the collection of urban and sometimes, (pre-treated) industrial effluents [57]. 

Although the treatment in WWTPs is followed by natural processes, such as sorption, photolysis and biodegradation, that can reduce the contaminant loads up to 10 times [58,59], the MPs’ presence is still unambiguous. In a research conducted among many countries, the most frequently encountered drugs were the non-steroidal anti-inflammatory drugs (NSAIDs), Sulfamethoxazole, Carbamazepine and Triclosan [2]. Generally, the occurrence of MPs was less frequent in summer (probably due to elevated, temperature-driven biodegradation), and even though winter rain promoted dilution, sometimes the contribution in natural water was important [60]. Finally, the concentrations found in surface waters were well correlated with the population distribution, linked with the massive utilization of parent chemicals by a bigger number of users [2].

Concerning drinking water, the studies are relatively few, because the occurrence is sometimes below the detection limit [60,61]. However, this is often a limitation of the experimental capabilities of the analytical laboratories. Kummerer has discussed this problem, in the appearance of “new” compounds, which could have been normally encountered (for pollutants in ng or μg scale) [62], if the technology allowed so. In addition, as far as long-term side-effects are concerned, the presence of certain compounds or their intermediated in drinking water has not been under study (yet). In overall, in the review published by Luo et al. [2] it is mentioned that most of the countries investigated (France, USA, Spain, and Canada) were capable of removing the presence of some MPs in drinking water. This is a critical step, considering that drinking water treatment is literally the last line of defense among end-users and micropollutants [33].

Although in developed countries water is considered a de facto supply in each household, in developing countries, the reality is sometimes far from this state. Water acquisition can be an everyday struggle for many families. If in this scenario, one thinks of the potential problems that could appear if MP pollution is high, the risks are more imminent. The quality of life of the affected population is considerably endangered, and more specifically not by chronic or potential problems, but from the harsh reality of raw, untreated wastewater in the water supplies. 

In many developing countries, the combination of rampant population growth and the lack of financial means, have led to insufficient (up to inexistent) sanitation facilities. Therefore, the collection and the treatment of wastewater is problematic. The poorest fractions of the population, who employ themselves in handcraft, fishing and agricultural activities suffer the most, since the situation in centralized, capital areas is slightly better. However, these areas have demonstrated unacceptable treatment, especially in (semi)industrial or hospital effluents, with cases describing direct untreated water being discharged in rivers and sea. 

“Fortunately”, the risk of MPs is relatively lower, when compared with developed countries. The availability of drugs and the capability of purchase restrict the widespread use and the diffuse pollution. The main areas expected to provide major MP flows are the hospitals and similar facilities. Recent research that has been performed in the framework of the “Treatment of the Hospital wastewaters in Ivory Coast and in Colombia by advanced oxidation processes” (unpublished data) indicated that even in this case, the majority of administered drugs are biodegradable and the MP content is limited in isolated hospitals in Colombia, but in the University Hospital in Abidjan, the situation is quite problematic.

On the other hand, even when the amounts of MPs is not alarming, the presence of microorganisms in WW is an important matter, which becomes top priority, since no disinfection process is applied in the effluents. Therefore, the focus should be directed at least to mitigating microbial pollution [63,64], when it comes to discharged WW in developing countries, which poses direct and acute illness risks. Hospitals are an identified contributor to fecal and overall pathogen microorganisms in surface waters, and the lack of treatment is directly jeopardizing their use [37]. The current practices in agriculture for instance, include the use of contaminated water for crops irrigation, and the transfer of pathogens is highly possible. Therefore, treatment designed taking account the local particular conditions and monitoring of total coliform bacteria and aerobic mesophilic bacteria, as representative of fecal and non-fecal routes, respectively, should be monitored and their elimination should precede discharge in natural water bodies [65,66].

## 5. Treatment Strategy and Research Results

**Figure 1:**
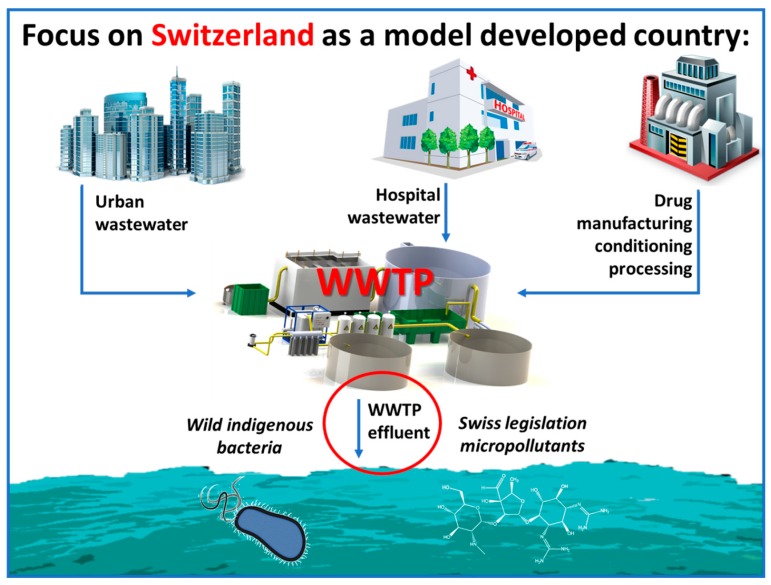
**Schematic representation: Treatment focus, strategy and targets of WWTP by light-assisted AOPs in developing countries.** Switzerland and its motion of upgrading the WWTPs is studied, presenting solutions based on the application of mainly UV-based AOPs, compared with the solar photo-Fenton. The targets are the micropollutants chosen by the Swiss evolution of control policy and the indigenous population of bacterial microorganisms present in WW.

As described in the previous sections, the issue of hospital wastewater treatment has multiple contextual, application and engineering extensions. The relevant literature has successful applications of various AOPs both as pre- and as post- biological treatment methods for hospital wastewater [67,68,69,70,71,72,73,74]. Our main goal here is to address the difference that the context of application can make in the choice and application of AOPs for hospital contaminants. The necessary strategies need to be specifically addressed towards developed or developing countries, where HWW is channeled in MWW or is directly discharged, respectively; a summary of the strategies is presented in Figure 1. The context differs significantly; the developed countries have more or less under control the problem of MOs and focus on MPs, while developing countries’ priority should be the acute risk caused by MOs presence. Furthermore, the AOP chosen has to be a function of the technical and economic status of the place of application, with the UV-based methods being more prominent in developed countries and the solar based ones more suitable for developing countries. As a result, one should take into account the aforementioned constraints when focusing on HWW treatment by AOPs in developed and developing countries, emphasizing both on the application point of view, as well as the underlying mechanisms governing micropollutant degradation and microorganism inactivation.

### 5.1. Developed Countries and Municipal WWTPs: Treatment of MPs and MOs by Light-Assisted AOPs

In Switzerland (as an example of developed country), the wastewater effluents are already treated, and the implementation of the relative AOPs promoted by the Federal Office for the Environment focuses on the elimination of the chronic risk caused by the presence of micropollutants in natural waters, and less for the acute risk of microbial infection due to the pathogens carried within the flows. As such, the micropollutants chosen derive from the modifications in the Swiss legislation, namely Carbamazepine, Clarithromycin, Diclofenac, Metoprolol, Venlafaxine, Benzotriazole and Mecoprop. Concerning microorganisms, the bacterial populations contained in urban effluents of the WWTP of Vidy, Lausanne were selected as microbial targets. 

**Figure 2:**
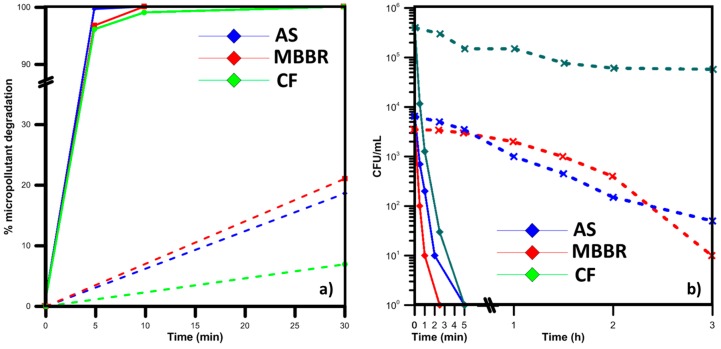
**Degradation of chemical and microbiological contaminants in MWW.** (**a**) Weighted average removal of seven selected micropollutants by the UV/H_2_O_2_ process (ng/L initial MP content and 25 mg/L H_2_O_2_); and (**b**) reduction of bacteria contained in WW by the UV/H_2_O_2_ (continuous trace) and the solar photo-Fenton process (dashed line). The colors correspond to the previous secondary treatment (blue: Activated Sludge, red: Moving Bed BioReactors, green: Coagulation-Flocculation). More details on the micropollutants’ initial content, experimental configuration and wastewater characteristics can be found in [78,79].

Figure 2 summarizes the results achieved by treating the effluents of Vidy WWTP by UV/H_2_O_2_ and the photo-Fenton process, where Figure 2a focuses on MPs and Figure 2b on bacterial microorganisms. The different color traces correspond to the different secondary pretreatment process applied in the plant before the lab-treatment by AOPs. 

The UV-based process is a far more energetic and therefore more efficient process for the removal of microbiological and chemical contaminants. The difference is in orders of magnitude higher, as a maximum 20% MP removal was attained by the photo-Fenton process in 30 min of exposure and at 10 min the corresponding removal by the UV/H_2_O_2_ process was 100% for activated sludge and moving bed bioreactor effluents (AS, MBBR) and 30 min were necessary for the coagulation-flocculation effluents (CF). Similar trends were found in microorganisms’ elimination, with 5 min exposure having completely eliminated microorganisms in all effluents, while more than 3 h were demanded by the photo-Fenton process. However, this process does not reflect the actual difference of the two processes, as increasing the Fenton reagents, for instance, would have a dramatic increase in efficiency, or, similarly, the H_2_O_2_ content in the UV-based process. Nevertheless, here we present only the mild conditions tested in lab-scale, but the literature has offered many successful applications of MP removals by the photo-Fenton process [75,76,77]. Apart from the reduction of micropollutants and microorganisms, a significant amount of effluent organic matter (EfOM) was eliminated from the bulk during treatment, reducing the overall charge carried before disposal [78]. 

The key in the application of either method is the economical and geographical context. For instance, there are very few countries that could support the application of such costly AOPs for domestic wastewater treatment; the estimated cost for the treatment plant of Vidy reaches 0.16–0.18 € m^3^ of treated waste for the application of activated carbon and ozonation [80]. The electrical cost and reagent (H_2_O_2_) supplies were calculated in a pilot plant tested in the same premises [81] and is of the same order. Nevertheless, this option is viable, considering that many plants in the USA operate chlorination basins, which are ideal for conversion to UV streams, and part of the cost is already administered and later will be recovered.

Concerning photo-Fenton, prolonging the treatment inflicts further degradation of contaminants and microorganisms, and a plant design with residence time of day(s) could be considered as an option. However, the main constraints under consideration are the land cost and the latitude of the site; beyond a certain point the clear/sunny days are decreased dramatically. The combination of these two factors on the other hand, apart from the developing countries who would greatly benefit from this process, already indicate USA and Australia as excellent candidates for application, especially due to high number of sunlit hours in Western USA and thorughout Australia. Even more, the application of maturation ponds is already an existing solution in the aforementioned regions, hence an addition of a polishing step aided by the photo-Fenton reagents could complete an already successful existing practice [82,83]. 

Finally, it was found that one of the most important concerns of wastewater treatment, the bacterial regrowth was sufficiently hindered [79]. For the UV-based processes, the complete elimination of microorganisms was attained by combined extensive mutations and hydroxylation of the cell membrane, which cannot be repaired by the enzymatic mechanisms possessed by microorganisms. The photo-Fenton process, even when it was not concluded, it did not present bacterial regrowth, owing to the presence of the Fenton reagents in the bulk; the continuous (dark) Fenton process ensures limitation of rampant bacterial growth and maintaining a good quality effluent in already inactivation was achieved. The design of the process should take into account both chemical and microbiological targets, since MPs require more time to degrade than bacteria, but bacteria engulf the regrowth risk, while the extended treatment times allow possible adaptation and growth in the rich WW medium. 

### 5.2. Developing Countries and Microorganisms Disinfection by Photo-Fenton

**Figure 3:**
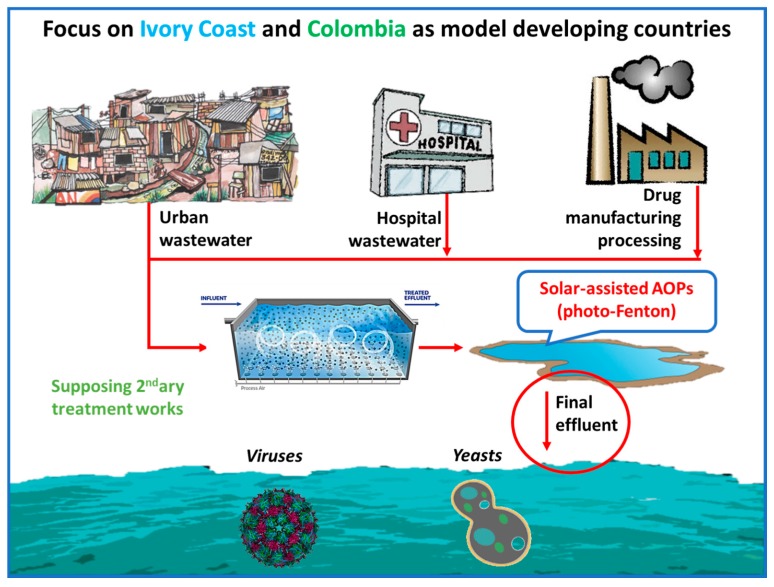
**Schematic representation: Using the photo-Fenton as a disinfection method in developing countries.** The application of Fenton reagents, aided by the numerous sunny days in the circum-Equatorial regions, which coincide with the geographical distribution of developing countries, was assessed on their virucidal and fungal inactivation capacities, using bacteriophages and common yeasts as proxies, while assessing the important role of dissolved organic matter and iron.

In Ivory Coast and Colombia, WWTPs are virtually inexistent. The practice of organized WW treatment is present only in major cities (e.g., Medellin), or in special establishments (hospitals in Ivory Coast; University Hospital in Abidjan). Furthermore, the treatment either stops at primary/secondary space, or has malfunctioned and not working properly since its construction. Therefore, the application of solar photo-Fenton as a feasible AOP is implied only after the construction of basic treatment before (primary-secondary), or as a possible implementation of a barrier of microbiological-related problems. The acute risk of microbial infections is prioritized over MPs, as it poses an immediate threat to the populations, and the assessment was made by taking a viral and a yeast pathogen model into study. A summary of the strategy is presented in Figure 3. 

Viruses are a special category of pathogens, responsible for a high number of disease outbreaks in developing countries. Their diversity and polymorphic nature make them an important target to consider when treating wastewaters. The MS2 bacteriophage model virus was chosen as it is particularly resistant to UV irradiation, hence its removal by solar-assisted processes could be delayed. As such, a step-wise construction was attempted, starting from solar light up to the photo-Fenton process. 

**Figure 4:**
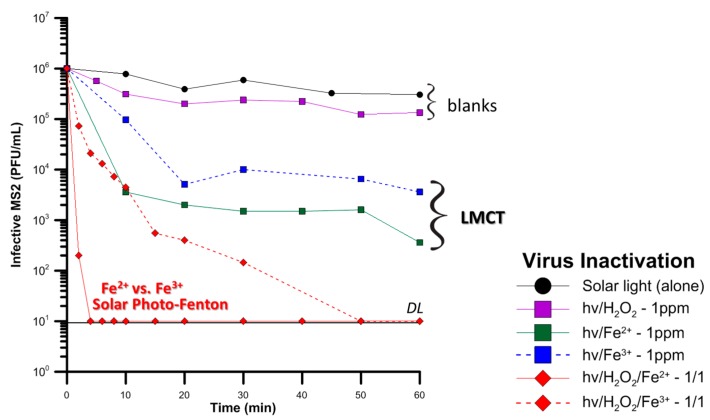
**Stepwise construction of the solar photo-Fenton process.** Effects of solar light, solar/H_2_O_2_, solar/Fe and solar photo-Fenton in viral (MS2) infectivity in simulated secondary wastewater, as in [87]. Note the difference inflicted by the initial speciation of iron in the solution.

Figure 4 presents the aforementioned approach on dissociating the events that take place during photo-Fenton treatment of MS2 coliphage. The step-wise construction was as follows:

(i) Solar light alone is unable to inflict high removal of MS2, hence underlines the need for application of an oxidative process.
(ii) Adding 1 mg/L H_2_O_2_, which is a moderate amount for inactivation of microorganisms, but could simulate the in-situ generation of H_2_O_2_ by irradiation of Dissolved Organic Matter (DOM) [84], again has almost no effect. The mild oxidative potential of H_2_O_2_ is unable to inactivate more than 1 log of MS2.
(iii) Continuing with the components of the photo-Fenton process, iron, in salts form, Fe^2+^ or Fe^3+^ was tested. Natural waters in Africa have often been found to contain high amounts of iron, especially in the form of oxides [85] but also dissolved. Although iron has no oxidative actions, its complexation with the viral capsid enables, upon irradiation, a LMCT reaction, with the reduction of Fe^3+^ to Fe^2+^ and oxidation of the ligand. This oxidation damages the external capsid proteins, thus reducing the infectivity of the virus.
(iv) The voluntary addition of very low amounts of H_2_O_2_ in the bulk indicate that the photo-Fenton process is potentially a very efficient treatment technique in the elimination of viral pathogens; the inactivation is either very sharp, or at least faster than the Fe or solar alone. Fe^2+^ is readily oxidizable, generating HO^•^ species, reaching viral inactivation, and Fe^3+^ after an initial reduction step, participates in the photo-Fenton catalytic cycle.
(v) Finally, the presence of organic matter in all the experiments did not appear to significantly hinder the inactivation of viruses. In fact, in presence of organic matter, the quantity of dissolved iron was followed, and the precipitation due to the neutral operating pH was avoided. Hence, a sustainable catalytic cycle can be maintained, aided by the iron-DOM complexes in WW [86].

For yeast-related experiments, *Saccharomyces cerevisiae* were used as a model of eukaryotic pathogen microorganisms. The strategy followed initially was similar to the virus inactivation, with stepwise construction of the inactivation process. On the other hand, the target was to study the internal events that take place during exposure to the photo-Fenton reagents. After initial experiments in simulated wastewater, where the inactivation of the yeast was normally achieved, the continuation involved tests only in pure water in order to avoid interferences with the genomic and proteomic measurements.

**Figure 5:**
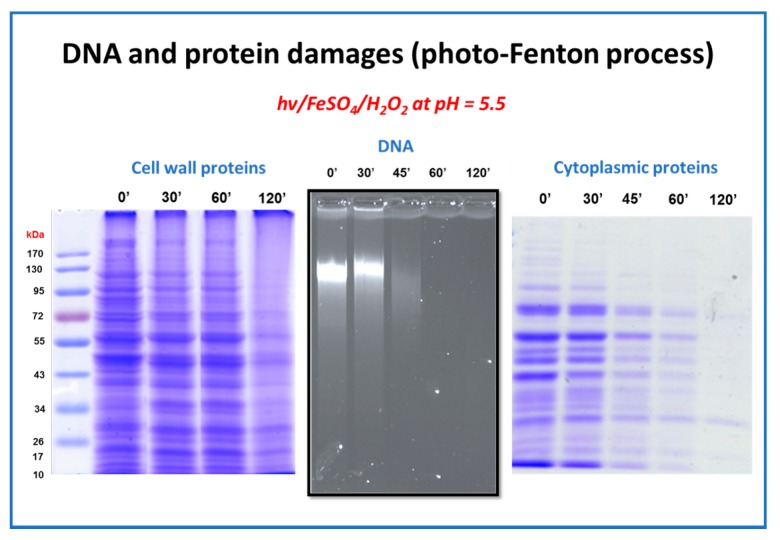
**Internal and external damages during *Saccharomyces cerevisiae* inactivation by the photo-Fenton process at near-neutral pH.** The panels indicate the external and two types of internal damage inflicted to the model yeast pathogen: cell wall proteins, genome and cytoplasmic proteins, respectively.

Figure 5 summarizes the results of the protein and DNA degradation experiments that took place during the photo-Fenton process. The events that take place during inactivation are inverse, compared to the expected series of events; one would suppose that since Fe and H_2_O_2_ are added in the bulk, the rapid reaction and generation of hydroxyl radicals would lead to the fast degradation of the (external) cell wall proteins. In fact this is partially true, as depicted in the first graph. The disappearance of the intensity of the bands during electrophoresis means that the proteins are degraded. However, if we correlate the damage taking place in 60 min, during which total disappearance of the DNA and heavy damages in the cytoplasmic proteins occurs, the slight fade in the cell wall protein bands is clearly not the main reason for yeast inactivation. 

As it appears, even in a complex, evolved microorganism such as the yeast, who possess repairing enzymatic functions, and a fortified (compared to bacteria) external cell wall, the Achilles’ heel is located in the internal part of the microorganism [88]. H_2_O_2_ can diffuse in to the cell, and with the lack of enzymes able to catalyze its dismutation to H_2_O and O_2_, due to sunlight exposure, the inactivation indeed takes place in the internal part. Even more, the iron that can be transported internally with various routes, or the already existing iron, released via the oxidative damage and the light-induced events, can facilitate an internal photo-Fenton process, able to inactivate yeasts. 

### 5.3. Hospital-Derived and Highly Concentrated Flows in Developed and Developing Countries: Iodinated Contrast Media and Drugs Treatment by Light-Assisted AOPs

**Figure 6:**
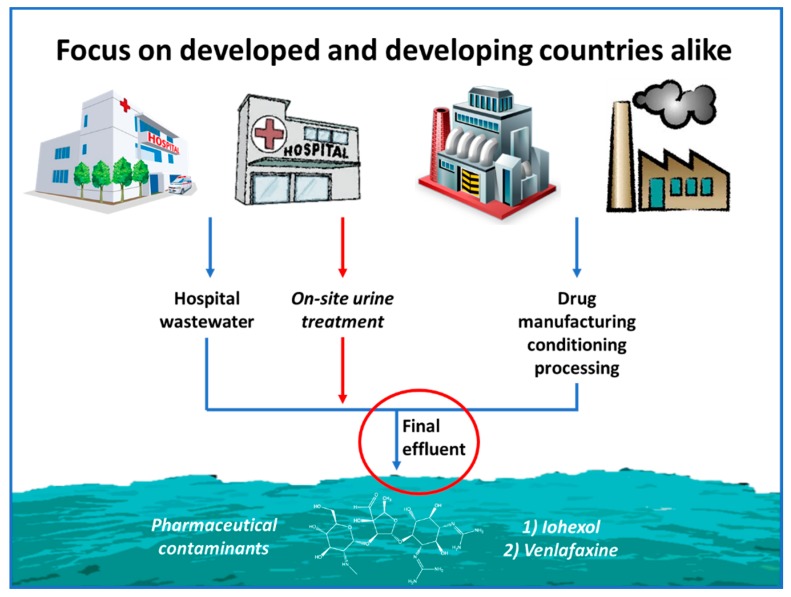
**Schematic representation: Treatment of hospital and highly concentrated flows of PhACs.** The treatment methods were associated with the concentration of the matrix (urine), hence UV-based solutions were sought, or the WW flows, allowing the assessment of photo-Fenton as a viable solution.

In both developed and developing countries, as mass flows of special drugs derive from hospital and production sites, two pollutants (Iohexol and Venlafaxine) in high amounts have been chosen and their degradation by relevant AOPs was studied. These drugs can be encountered in the production wastewaters or in urine, due to the treatment of patients, and the (pre)treatment of concentrated flows at hospital or manufacturing level is desirable before dilution in the municipal wastewater streams. A summary of the strategies can be found in Figure 6.

**Figure 7:**
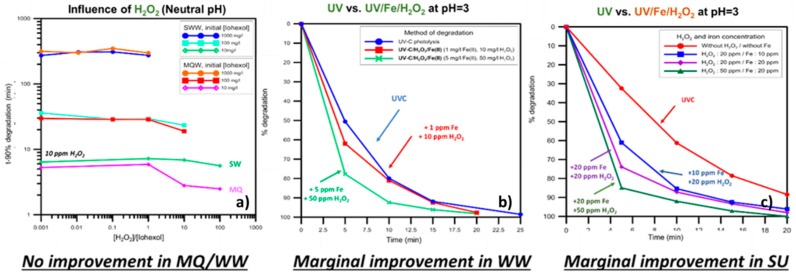
**ICM Iohexol degradation by UV-based AOPs in:** (**a**) **water;** (**b**) **wastewater; and** (**c**) **urine.** H_2_O_2_ addition and Fe-assisted experiments, aided by acidification of the matrix hold minor improvement in degradation kinetics.

The Iodinated Contrast Medium (ICM) Iohexol is characterized by its recalcitrance to biological treatment, since its medical prescription aims to stay unchanged in the body for the duration of the tests of the patient. As such, it leaves the body with the same composition, making its discharge a problem; the doses for various exams may reach grams and was equally detected in Swiss and Ivorian HWW flows. In Figure 7, it is shown that applying UVC irradiation to spiked water, (simulated) wastewater and urine samples was very efficient in removing the parent compound, giving rise to de-iodinated intermediates that remained in the bulk, with no total organic carbon (TOC) reduction. Enhancing its action with H_2_O_2_ and by the production of HO^•^, did not significantly enhance the degradation kinetics, however moderately reduced the TOC of the solution, thus removing some of the recalcitrant intermediates. Similarly, adding iron to enhance the action of UV/H_2_O_2_ by the parallel UV-Fenton process only slightly enhanced the degradation kinetics, however the analysis of the intermediates revealed pathways that were not achieved during the degradation of the parent compound by UVC alone [89]. 

Another highlight of the investigation was the opportunity presented to treat urine contaminated with Iohexol. By simulating the immediate collection of urine from the patient, it was shown that the contamination levels, can be significantly reduced before the discharge and subsequent dilution of the urine in HWW or UWW collection systems. The economic gain of such process would impact the treatment necessities of the final plant, e.g., AOPs, after secondary treatment. Hence, UV-based AOPs can play also a role of pre-treatment for Iodinated Contrast Media-containing effluents. Finally, although the use of iron was not justified by the kinetic investigation, the acute fractionation of the compound could be correlated with the opportunity to pre-treat such drugs prior to biological treatment, which is significantly cheaper from the AOPs treatment. 

**Figure 8:**
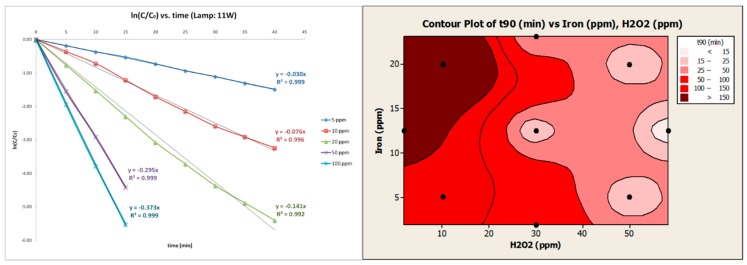
**Antidepressant Venlafaxine degradation in MQ water.** (**a**) UV/H_2_O_2_ mediated degradation and kinetic evaluation of H_2_O_2_ incremental addition; and (**b**) solar photo-Fenton at acidic pH, with the optimal degradation areas (light color contour).

The other drug, Venlafaxine, is a compound that belongs in the broad family of serotonin-norepinephrine reuptake inhibitors, and is widely prescribed as an antidepressant. Although the amounts given are nothing like the ones encountered in the case of Iohexol, the results of the exposure of aquatic organisms in this drug has shown severe side-effects, such as alteration of the predation behavior of fish and disrupted locomotion of invertebrates [90,91,92]. Its treatment has been assessed by AOPs as TiO_2_ photocatalysis and was further achieved by several AOPs, including UV-based and solar-assisted ones [93,94]. In Figure 8, the kinetic analysis showed a drug that can be removed without resolving to extreme conditions; moderate H_2_O_2_ addition (UVC light or few mg/L Fe, either combined with 10–25 mg/L) and reasonable residence time was necessary to remove the parent compound. However, the intermediate degradation was delayed revealing a recalcitrant nature of the drug. Nevertheless, its degradation was achieved in aqueous matrices such as MQ water, WW (real secondary and synthetic one), leaving promise for its efficient elimination in UWW or HWW. However, even in urine, which is a highly loaded matrix, with high UV absorbance, quantities as low as few μg/L were efficiently removed as followed by UPLC/MS analyses. 

Although the kinetics are important when its degradation is considered in MWTPs, other remarkable observations were made, with application in HWW or industrial-level (mg/L) contaminated waters. The pretreatment with UV-based or solar-assisted AOPs enhanced the biodegradability of the treated effluent, compared to the initial one. The gold standard of biodegradability tests, the Zahn–Wellens test revealed up to 20% increase in biodegradability of the treated effluents, by UV/H_2_O_2_ or solar photo-Fenton [95], in simulated WW containing mg/L amounts of the selected pharmaceutical. Hence, the restriction of dilution of HWW or manufacturing flows could be effectively pre-treated. 

## 6. Conclusions

The use of light-assisted AOPs towards pollutant decontamination and disinfection of effluents, namely UV, UV/H_2_O_2_, solar light (shown to work as an AOP), Fenton and solar photo-Fenton are established as powerful allies in the ongoing task of wastewater purification. The key conclusions can be summarized as follows:

(1) UV-based AOPs are efficient for MP removal and MO inactivation. Although changing dynamically, the Swiss reality on hospital wastewater treatment dictates their discharge in the municipal collection network, and therefore implies their co-treatment with municipal wastes. The UV-based AOPs (UV and UV/H_2_O_2_) were found to be effective micropollutant removal strategies in ng/L level and bacterial inactivating processes, after biological secondary pre-treatment, as found in municipal wastewaters. When used in simulated hospital wastewaters and urine treatment, as alternative micropollutant elimination strategies, their efficiency was measured and established against a list of contaminants, with parallel elimination of the contained organic matter. The degradation was fast, and the reactants addition and necessary light doses were moderate.
(2) The solar photo-Fenton process and its constituents can be very effective in the proper context. Despite the lower apparent efficiency of this process when compared with its UV-based counterparts, photo-Fenton was found to effectively and non-selectively remove micropollutants and effluent organic matter. Furthermore, their application resulted in high bacterial removal, regrowth suppression, and yeasts and viruses inactivation from water and wastewater effluents. Most importantly, through systematic studies the mechanism and the key points of the process against the aforementioned targets were characterized. Special emphasis was given to the organic matter present in WW, as it is found to hinder the inactivation process but other benefits, such as iron complexation, also occur.
(3) The selected model hospital/industrial contaminants (Iohexol, Venlafaxine) helped elucidate the pitfalls and opportunities in HWW treatment by AOPs. The AOPs were found to work particularly well against the concentrated, (simulated) industrial wastewater, hospital flows and urine. Therefore, their application in hospitals and related industrial activities is promising. In addition, the structural deformation of the selected pollutants provided helpful insights on the operational and chemical constraints on applying the various AOPs; for instance the use of iron (when H_2_O_2_ is present) is strongly recommended for faster and more intense degradation of the contaminants in HWW. Finally, apart from the degradation point of view, the AOPs studied increased the biodegradability of the selected compounds treated solutions, which could allow their use as pre-treatment methods in HWWTPs.

In conclusion, more work is necessary to establish these methods as suitable for application in hospital environments. However, the initial results strongly support their further development, and future work stemming from the present research is encouraged to be sought.

## Figures and Tables

**Table 1:** Comparison between indicative MWW and HWW effluents characteristics [31,35,36] and references therein.

Parameter	Details	Municipal WW	Hospital WW	Units
BOD		60	160	mg/L
COD		110	280	mg/L
SS		80	135	mg/L
pH		7.5	8	
TKN		20–70	33	mg/L
Chlorides		50	200	mg/L
Total P		4	7	mg/L
Bacteria	*Total Coliforms*	10^6^	7.7 × 10^9^	CFU/mL
Viruses	*Norovirus*	1.6 × 10^2^	2.4 × 10^6^	PFU/mL
	*Hepatitis A virus*	10^2^	10^4^	PFU/mL
	*Adenovirus*	1.6 × 10^2^	2.8 × 10^6^	PFU/mL

## Abbreviations

Activated Sludge (AS), Advanced Oxidation Processes (AOPs), Biochemical Oxygen Demand (BOD), Chemical Oxygen Demand (COD), Coagulation-Flocculation (CF), Dissolved Organic Matter (DOM), Hospital wastewater (HWW), Iodinated Contrast Medium (ICM), Ligand-to-Metal Charge Transfer (LMCT), Microorganisms (MOs), Micropollutants (MPs), Moving Bed Bioreactor (MBBR), Municipal Wastewater (MWW), Non-Steroidal Anti-Inflammatory drugs (NSAIDs), Pharmaceutically Active Compounds (PhACs), Reactive Oxygen Species (ROS), Suspended Solids (SS), Total Organic Carbon (TOC), Wastewater (WW), Wastewater treatment plant (WWTP).
